# Surgical Fixation of Calcaneal Beak Fractures—Biomechanical Analysis of Different Osteosynthesis Techniques

**DOI:** 10.3389/fbioe.2022.896790

**Published:** 2022-08-04

**Authors:** Martin C. Jordan, Lukas Hufnagel, Miriam McDonogh, Mila M. Paul, Jonas Schmalzl, Eva Kupczyk, Hendrik Jansen, Philipp Heilig, Rainer H. Meffert, Stefanie Hoelscher-Doht

**Affiliations:** Julius-Maximilian-University of Würzburg, Würzburg, Germany

**Keywords:** foot, ankle, Achilles, tendon, fracture

## Abstract

The calcaneal beak fracture is a rare avulsion fracture of the tuber calcanei characterized by a solid bony fragment at the Achilles tendon insertion. Treatment usually requires osteosynthesis. However, lack of biomechanical understanding of the ideal fixation technique persists. A beak fracture was simulated in synthetic bones and assigned to five different groups of fixation: A) 6.5-mm partial threaded cannulated screws, B) 4.0-mm partial threaded cannulated screws, C) 5.0-mm headless cannulated compression screws, D) 2.3-mm locking plate, and E) 2.8-mm locking plate. Different traction force levels were applied through an Achilles tendon surrogate in a material-testing machine on all stabilized synthetic bones. Outcome measures were peak-to-peak displacement, total displacement, plastic deformation, stiffness, visual-fracture-line displacement, and mode of implant failure. The 2.3- and 2.8-mm plating groups showed a high drop-out rate at 100 N tension force and failed under higher tension levels of 200 N. The fracture fixation using 4.0-mm partial threaded screws showed a significantly higher repair strength and was able to withhold cyclic loading up to 300 N. The lowest peak-to-peak displacement and the highest load-to-failure and stiffness were provided by fracture fixation using 6.5-mm partial threaded cannulated screws or 5.0-mm headless cannulated compression screws. As anticipated, large 6.5-mm screw diameters provide the best biomechanical fixation. Surprisingly, the 5.0-mm headless cannulated compression screws yield reliable stability despite the absent screw head and washer. When such large screws cannot be applied, 4.0-mm screws also allow reasonable fixation strength. Plate fixation should be implemented with precaution and in combination with a restrictive postoperative motion protocol. Finally, clinical cases about the surgical application and recovery are included.

## Background

**FIGURE 1:**
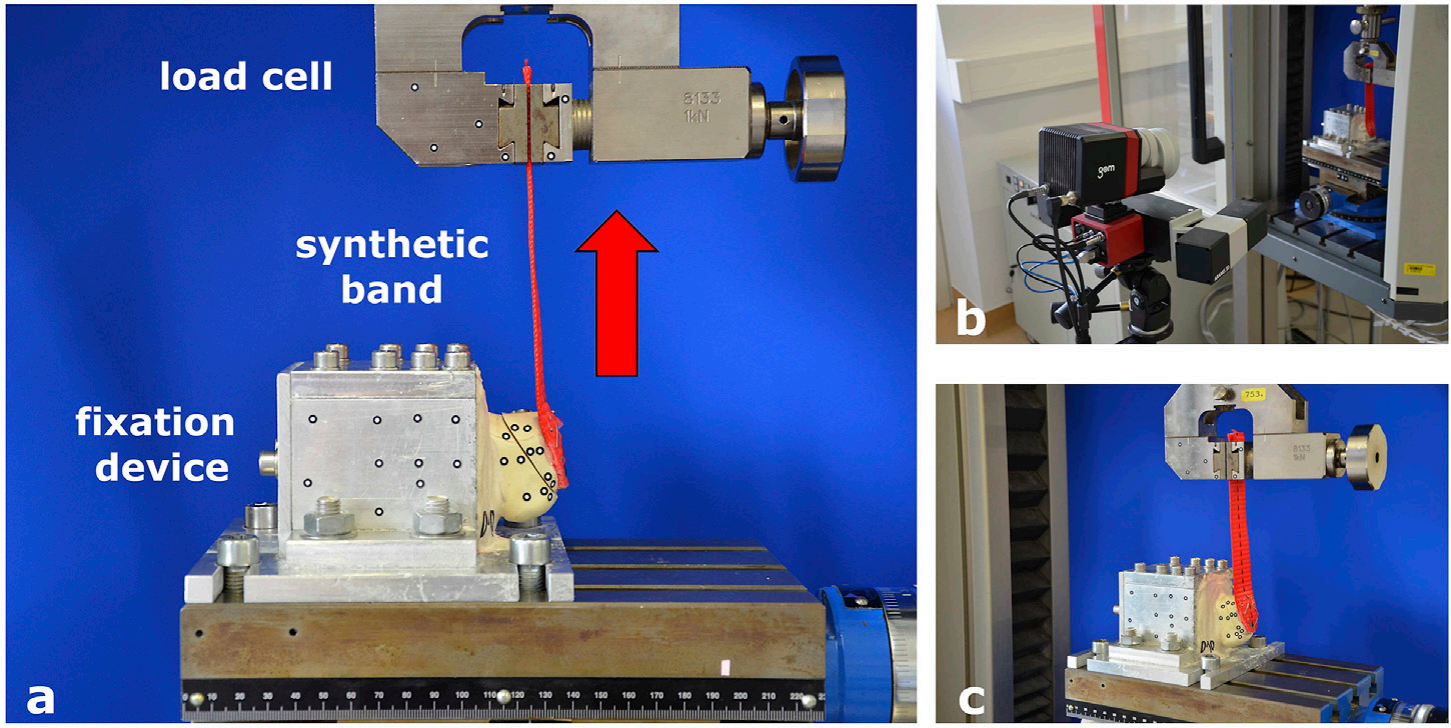
Test set-up and fixation in the material testing machine. **(A)** Fixation device for synthetic bone specimens to the material testing machine. **(B)** Optical measuring machine. **(C)** Band mimicking the Achilles tendon to apply tension to the fragment.

**FIGURE 2:**
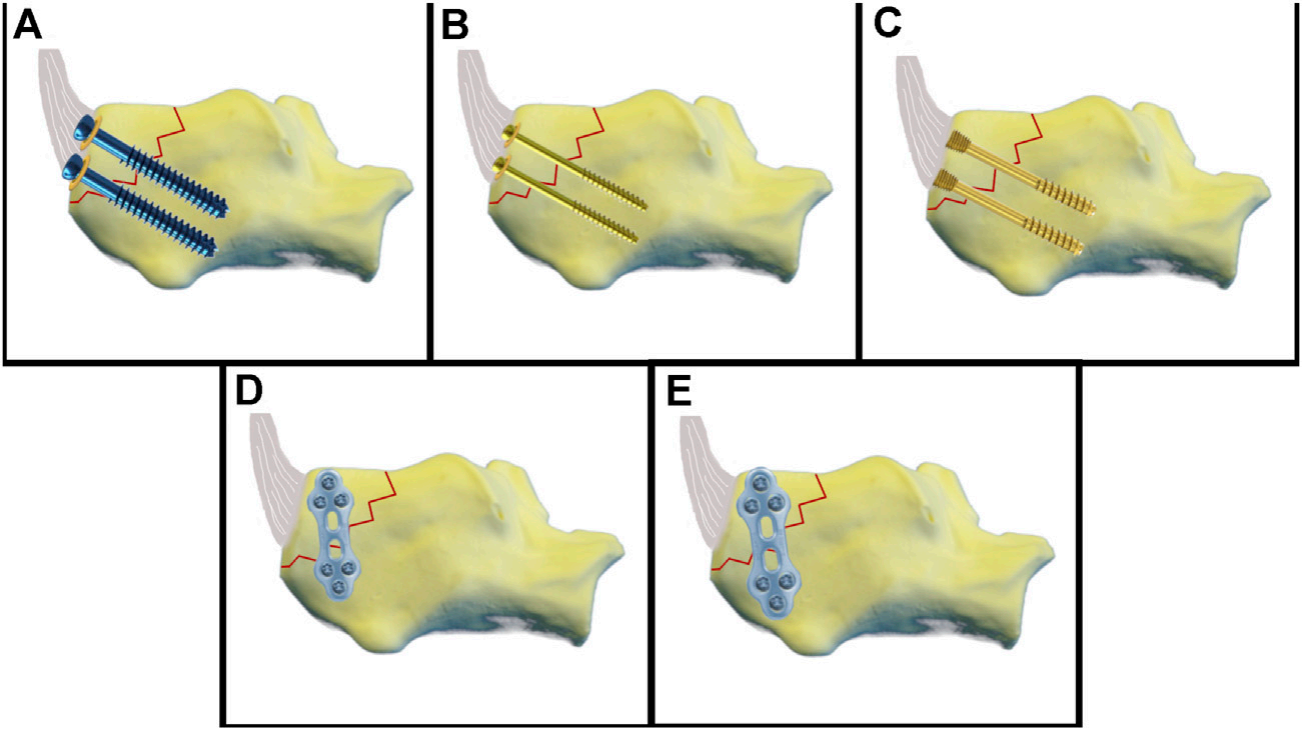
Schematic illustration of the fixation techniques. **(A)** 6.5-mm partial threaded cannulated screw. **(B)** 4.0-mm partial threaded cannulated screw. **(C)** 5.0 headless cannulated compression screw. **(D)** 2.3-mm plate fixation. **(E)** 2.8-mm plate fixation.

The beak fracture is a rare calcaneal fracture subtype of the posterior calcaneal tuberosity (Warrick and Bremner, 1953; Carnero-Martín de Soto et al., 2019). The available data indicate that elderly patients with osteopenic or osteoporotic bones are more likely to be affected by this fracture (Carnero-Martín de Soto et al., 2019; Beavis et al., 2008; Lee et al., 2012). Beavis et al. (2008) described three different fracture types based on the extent to which the tendon insertion is affected at the tuber calcanei. In type I fractures, a shell of bone avulses from the posterior tuberosity. Type II describes fractures with a solid bone fragment, where an oblique fracture line runs toward the posterior end of the posterior facet (Figures 1, 2, and Figure 7A). Type III fractures are infrabursal avulsions from the middle third of the posterior tuberosity. The injury itself is usually the result of sudden and disproportional muscular contractions, where the Achilles tendon rips a solid bony fragment out of the tuberosity. These fractures require urgent treatment because the pressure on the thin soft tissue coverage can cause severe necrosis (Banerjee et al., 2012; Mitchell et al., 2019). The small osseous fragments in type I and III fractures are typically refixed using suture anchors or transosseous sutures (Banerjee et al., 2012; Wakatsuki et al., 2016). For type II fractures, the literature recommends open reduction and fixation using 4.5- or 6.5-mm partially threaded screws (Banerjee et al., 2012; Gitajn et al., 2015). What remains unclear, however, is which type of screw is least likely to result in complications such as screw pull-out or screw cut-out (Banerjee et al., 2012; Lee et al., 2012; Carnero-Martín de Soto et al., 2019). The purpose of this study was to perform a comprehensive biomechanical comparison of currently available operative fixation techniques and to demonstrate their application in selected clinical cases. Given the high potential of failure with some of these methods, this study should help elucidate the most reliable technique (Gitajn et al., 2015).

## Materials and Methods

### Specimens and Fracture Generation

A total of 50 synthetic bone specimens of the calcaneus (LD 9118; Synbone, Zizers, Switzerland) were used in this study. Previous studies have shown that the biomechanical properties of synthetic bone and human specimens are similar, and our pre-tests confirmed these findings. A comparison of different synthetic bone models showed that Synbone most closely mimics the bone structure of elderly patients (Hoelscher-Doht et al., 2014; Fuchs et al., 2020). A Beavis type II fracture was induced using an oscillating saw. The size of the triangular fragment was 2.0 × 3.2 × 4.2 mm. The Achilles tendon was simulated by a braided synthetic band (kwb Germany GMBH, LC 1500 daN, Art.-Nr. 772,395) attached to the fragment (EPO-X-Y, Roxolid, Germany and staples).

### Test Set-Up

A custom-made aluminum fixation device was developed to fit into the testing machine. Two-thirds of the bone specimen was embedded in the fixation device using calcium sulfate Ca [SO_4_] 2H_2_O, leaving the tuber calcanei free. The fixation device was mounted to the bottom of the testing machine, and the synthetic band simulating the Achilles tendon was affixed to a clamp connected to the load cell. Visual markers for video capturing were placed on the synthetic bone, with six markers on either side of the fracture. More markers were attached to the fixation device as reference points (Figure 1).

### Experimental Groups

**FIGURE 3:**
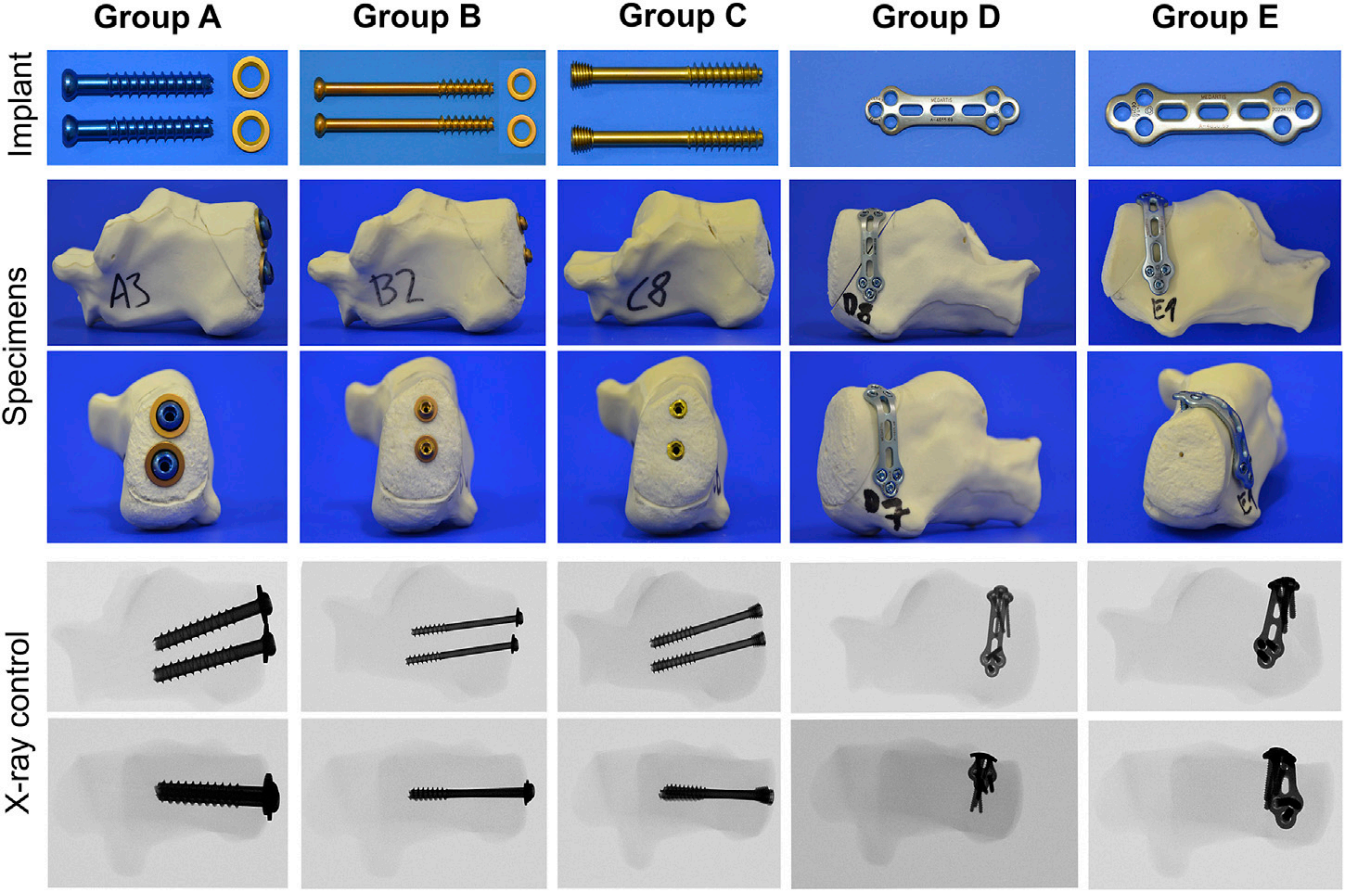
Overview of the fixation techniques tested, including the implant material, stabilized synthetic bones, and fluoroscopic imaging.

The fragment in Group A was fixed using two cannulated 6.5 mm threaded screws with underlying washers (length 45 mm, REF 408.431, b7, DePuy Synthes, Johnson & Johnson, United States). First, the fracture was reduced and fixated with two 2.8 mm k-wires. Using a cannulated 5.0 mm drill bit, the cannulated screws were inserted over the k-wires and underlaid with round washers (REF 419.990). Group B was stabilized with two cannulated 4.0 mm partially threaded screws and washers (length 44 mm, REF 407.644, DePuy Synthes). The fracture was reduced and fixed with two 1.25 mm k-wires. Using a cannulated 2.7 mm drill bit, the cannulated screws and washers (REF 419.980) were inserted over the k-wires. Group C was fixed with two 5.0 mm headless cannulated compression screws (length 45 mm, A-8211.45X, Medartis). Headless cannulated compression screws were inserted *via* priorly placed k-wires until the screw head was buried on the bone level. Group D was fixed by a lateral 2.0/2.3 mm locking plate (TriLock Grid Plate 3 + 3 hole, 37 mm, t1.3, APTUS, REF A-4655.69, Medartis). Reposition was achieved by a pointed reduction clamp and temporarily fixed by k-wires. Bending pliers were used to contour the plate. Locking screws from 14–20 mm were used. Group E was stabilized by a lateral 2.8 mm locking plate (2.8 TriLock Grid Plate 3 + 3 hole, 43 mm, t1.6, APTUS, REF A-4850.69, Medartis) and locking screws (14–20 mm) (Figures 2, 3, and Table 1). In each group, ten synthetic bone specimens of the calcaneus were used.

**TABLE 1:** Different groups tested.

Group	Fixation	Implant	Company
A	2 × cannulated screws 6.5 mm and washer	Length 45 mm, partial threaded, REF 408.431; washer 13 mm, REF 419.990	DePuy Synthes
B	2 × cannulated screws 4.0 mm and washer	Length 44 mm, partial threaded, REF 407.644; washer 7 mm, REF 419.980	DePuy Synthes
C	2 × 5.0 mm headless cannulated compression screws	Length 45 mm, REF A-8211.45X	Medartis
D	Plate fixation 2.3 mm	2.0/2.3 TriLock GridPI 3 + 3 hole, 37 mm, t1.3, APTUS, REF A-4655.69 + 2 × 16 mm + 2 × 18 mm + 2 × 20 mm locking screws	Medartis
E	Plate fixation 2.8 mm	2.8 TriLock Grid PI 3 + 3 hole, 43 mm, t1.6, APTUS, REF A-4850.69 + 2 × 14 mm + 2 × 16 mm + 2 × 20 mm locking screws	Medartis

### Biomechanical Protocol

Testing was conducted using a material-testing machine (Zwick/Roell Z020; Zwick GmbH & Co., Ulm, Germany) and the corresponding software (testXpert version 3.6; Zwick/Roell). The test protocol was determined according to our own pretests (load range 10–400 N; number of test cycles 10–8000). The final test protocol encompassed a 10 N preload followed by 10 setting cycles between 10 and 40 N. Following this, the test started with a cyclic loading from 10 to 100 N for 1,000 repetitive cycles. The next load level was 10–200 N for 1,000 cycles, and the third load level was 10–300 N for 1,000 load repeats: a static “ultimate strength” test was performed after cyclic testing. This measured load-to-failure and failure mode. For optical 3D metrology, a camera system (Pontos live, GOM, Germany) was placed in front of the material testing machine. The integrated software (Correlate Professional, 2018; GOM) captured marker displacement. The most caudally positioned marker on the fragment was used for visual analysis of the displacement. Parameters measured were *peak-to-peak displacement* for 100, 200, and 300 N in mm, *stiffness* (N/mm) and *plastic deformation* (mm), *total displacement* (mm), *load-to-failure* (N), and *mode of failure* (anterior fracture, caudal screw cut-out and cranial screw pull-out, caudal screw cut-out, caudal and cranial screw pull-out, pull-out of the fracture-fragment from the caudal screw, pull-out of the fracture fragment from both screws, fracture of the fracture-fragment at the caudal screw, fracture of the fracture fragment at the site of the cranial screws, and plastic deformation of the plate).

### Statistical Analysis

Microsoft Excel 2010 (Microsoft Corp. Redmond, WA, United States) was used for data collection. The data were analyzed using SPSS Statistics 27/28 (IBM Corp. Armonk, NY, United States). A power analysis was performed in previous tests using a power of 80% and a significance level of 5%, which showed that the sample size was adequate. The results are presented as the mean with standard deviation. All data were statistically analyzed for normal distribution using the Shapiro–Wilk test. Normally distributed data were compared using analysis of variance and the Bonferroni correction. Non normally distributed data were analyzed using the Kruskal–Wallis test and Dunn–Bonferroni correction. A *p*-value of <0.05 was considered statistically significant.

## Results

Results were grouped into biomechanical data gained by the material testing machine and visual data by the optical system.

### Drop-Out

Specimens in groups D and E were not able to bear the 200 and 300 N tensions and, therefore, could not progress to load-to-failure tests. Three specimens in Group D and one in Group E lasted the 1,000 cycles at 100 N. This rendered a sufficient statistical analysis of these groups impossible. Drop-out occurred during the cyclic loading at 300 N for one specimen in Group A, three specimens in Group B, and one specimen in Group C.

### Peak-to-Peak Displacement

**FIGURE 4:**
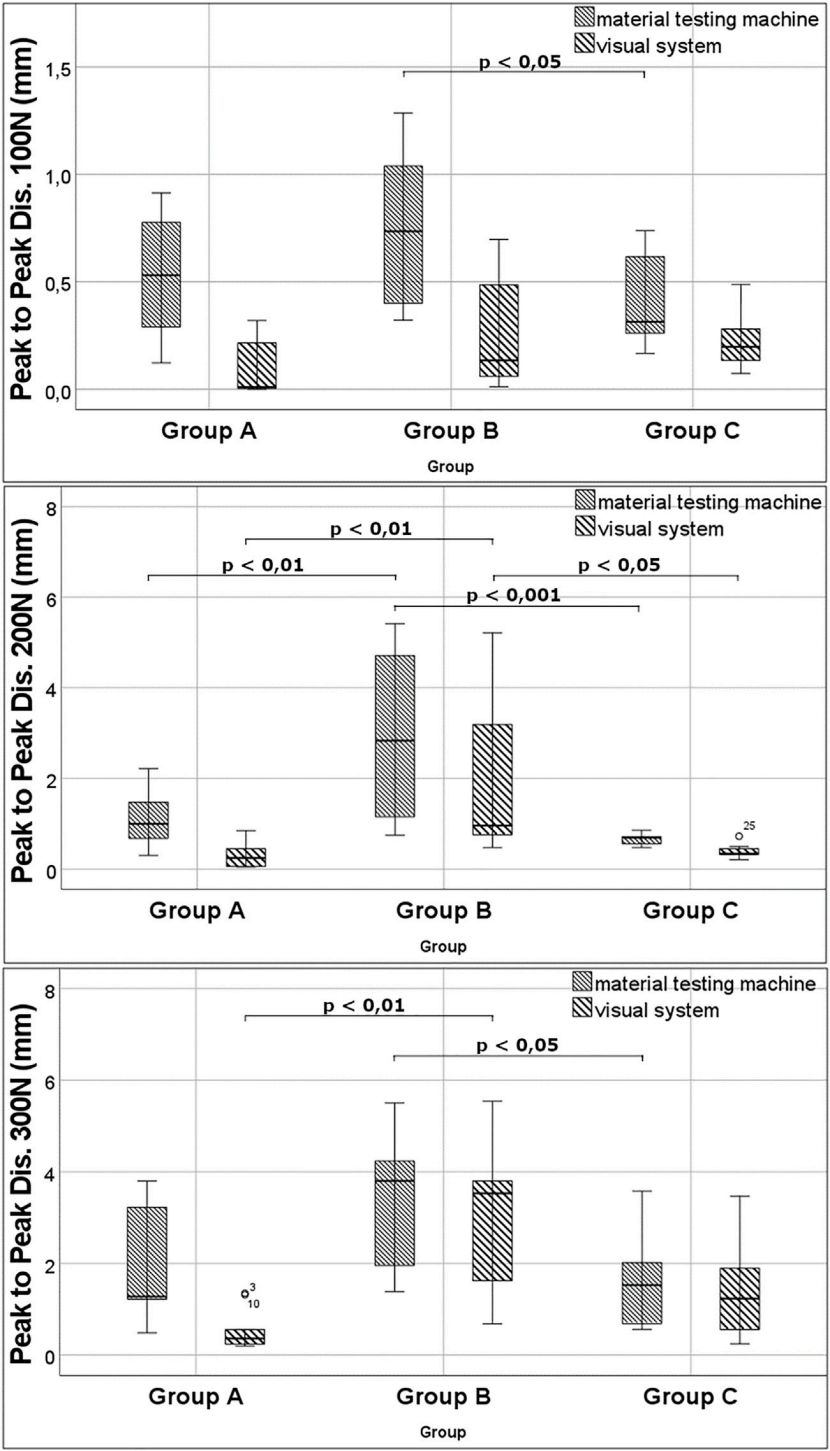
Results for peak-to-peak displacement at different load levels.

Biomechanical data: Means at the load level of 100 N were 0.5 ± 0.3 mm in Group A, 0.8 mm ± 0.4 mm in Group B, and 0.4 mm ± 0.2 mm in Group C. There was a significant difference between Group B and C with *p* = 0.023. For 200 N, means were 1.1 ± 0.6 mm in Group A, 3.0 ± 1.8 mm in Group B, and 0.7 ± 0.1 mm in Group C. At this level, statistical differences could be seen between groups A and B with *p* = 0.002 and between groups B and C with *p* < 0.001. For cyclic testing at 300 N, means were 1.8 ± 1.2 mm in Group A, 3.3 mm ± 1.6 mm in Group B, and 1.6 ± 1.0 mm in Group C. Data showed a significant difference between groups B and C with *p* = 0.048. Visual data: Means at the load level of 100 N were 0.09 ± 0.12 mm in Group A, 0.26 ± 0.25 mm in Group B, and 0.22 ± 0.13 mm in Group C. At this level, there was no statistical difference. Means for cyclic testing at 200 N were 0.30 ± 0.26 mm in Group A, 1.99 ± 1.66 mm in Group B, and 0.38 ± 0.15 mm in Group C. Significant differences could be seen between groups A and B with *p* = 0.001 and between groups B and C with *p* = 0.016. For the load level of 300 N, the means were 0.56 ± 0.45 mm in Group A, 2.94 ± 1.73 mm in Group B, and 1.34 ± 1.06 mm in Group C. The difference between groups A and B was statistically significant, with *p* = 0.008 (Figure 4).

### Total Displacement

Biomechanical data: the means were 3.9 ± 1.3 mm in Group A, 6.8 ± 1.5 mm in Group B, and 4.4 ± 1.2 mm in Group C. Statistical analysis showed significant differences between groups A and B with *p* = 0.001 and between groups B and C with *p* = 0.007. Visual data: the means were 1.11 ± 0.731 mm in Group A, 5.31 ± 2.94 mm in Group B, and 3.04 ± 1.44 mm in Group C. There was a significant difference between groups A and B with *p* = 0.002.

### Plastic Deformation

For the load level of 100 N, means were 0.1 ± 0.1 mm in Group A, 0.3 ± 0.2 mm in Group B, and 0.2 ± 0.1 mm in Group C. There was no significant difference. At 200 N load, the means were 0.4 ± 0.3 mm in Group A, 2.1 ± 2.1 mm in Group B, and 0.5 ± 0.2 mm in Group C. Data showed a significant difference between groups A and B with *p* = 0.013. Means for cyclic testing at 300 N were 0.7 ± 0.6 mm in Group A, 3.1 ± 2.3 mm in Group B, and 1.4 ± 1.1 mm in Group C. There was a significant difference between groups A and B with *p* = 0.006.

### Stiffness

**FIGURE 5:**
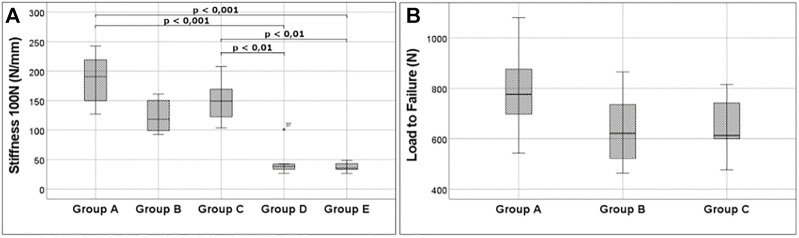
Important outcome. **(A)** Stiffness at 100 N for all groups analyzed. A very low stiffness is noticeable for groups D and E, underlining the weakness of plating. **(B)** Boxplots show the high fixation strength of 6.5-mm partial threaded cannulated screw screws. 5.0 headless cannulated compression screw, and 4.0-mm partial threaded cannulated screw can also resist high tension forces.

At a load of 100 N, means were 185 N/mm ± 42 N/mm in Group A, 124 N/mm ± 28 N/mm in Group B, 148 N/mm ± 31 N/mm in Group C, 44 N/mm ± 23 N/mm in Group D, and 37 N/mm ± 7 N/mm in Group E. Statistical analysis showed differences between groups C and E with *p* = 0.006, between groups C and D with *p* = 0.006, between groups A and E with *p* < 0.001, and between groups A and D with *p* < 0.001. Means at 200 N were 206 N/mm ± 48 N/mm in Group A, 121 N/mm ± 26 N/mm in Group B, and 151 N/mm ± 29 N/mm in Group C. There was a significant difference between groups A and B with *p* = 0.002. For cyclic testing at 300 N, the means were 202 N/mm ± 25 N/mm in Group A, 114 N/mm ± 15 N/mm in Group B, and 134 N/mm ± 22 N/mm in Group C. The data showed significant differences between groups A and B and groups A and C with *p* < 0.001 (Figure 5).

### Load-to-Failure

Means were 787 ± 184 N in Group A, 638 N ± 147 N in Group B, and 651 N ± 113 N in Group C. No significant difference was found for the maximum load (Figure 5).

### Implant Failure

**FIGURE 6:**
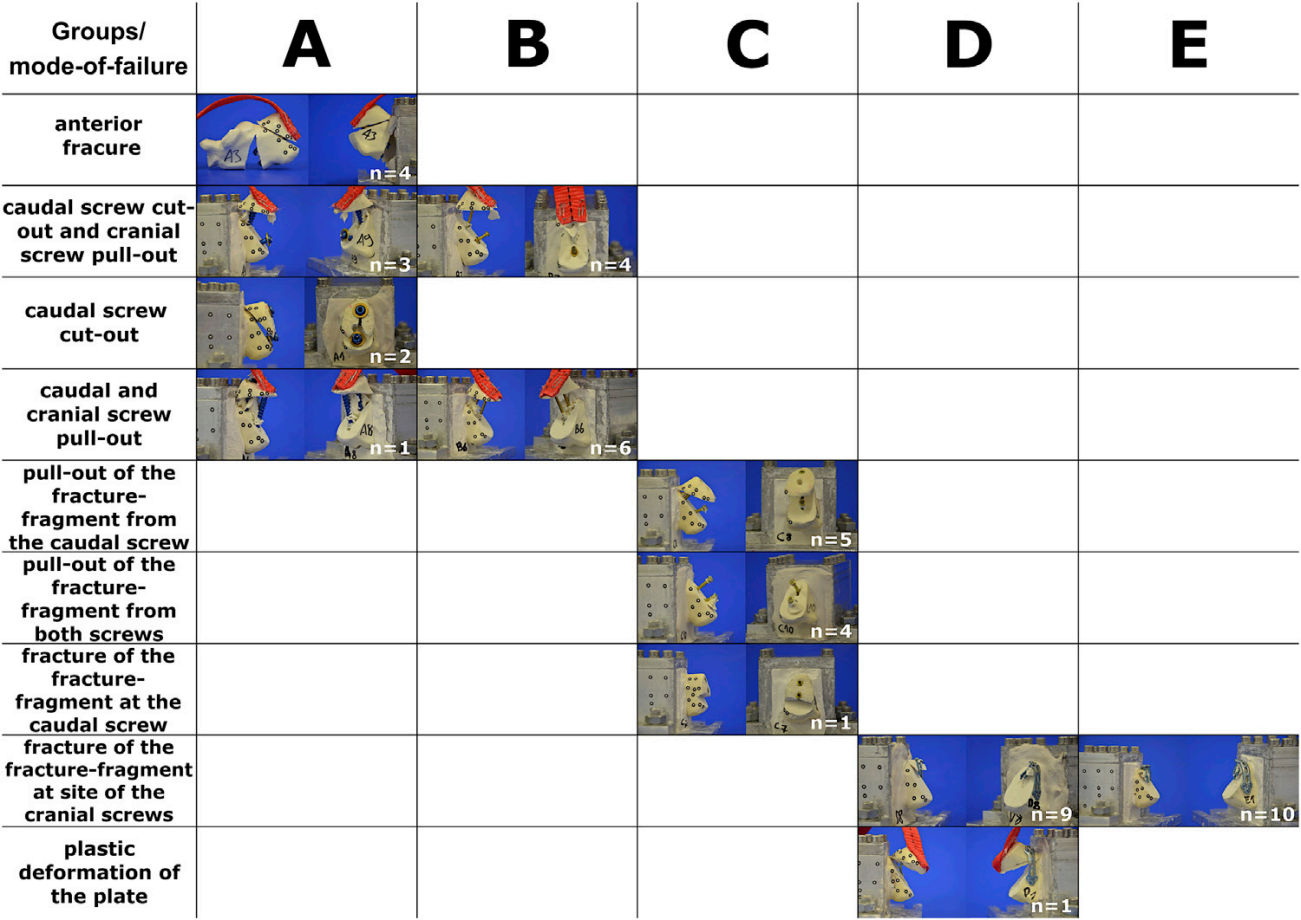
Type of implant failure sorted by groups. The horizontal column represents the groups tested **(A–E)**. The vertical column describes the different modes of failure. Please see also Supplementary Material at the end.

In Group A, four specimens developed an anterior fracture at the screw ends. Three specimens failed by a simultaneous cut-out of the caudal screw and pull-out of the cranial screw. In two cases, cut-out of the caudal screw occurred without pull-out of the cranial screw. One specimen failed by pull-out of both the caudal and cranial screws. In Group B, six specimens failed through the pull-out of both screws. Four specimens sustained a cut-out of the caudal screw and a pull-out of the cranial screw. In Group C, modes of failure were different to the ones described for groups A and B. Five specimens failed by pull-out of the fracture-fragment from the caudal screw. In four specimens, pull-out of the fracture-fragment occurred in both the caudal and cranial screw. One specimen failed by fracture at the level of the caudal screw. In Group D, almost all specimens failed by breakout of the fragment at the site of the screws. Failure due to plate deformation was only observed once. In Group E, the same mode of failure could be seen as in the majority of Group D. All objects failed by breakout of the fragment at the site of the screws (Figure 6; Supplementary Video S1).

## Discussion

Our data confirm the biomechanical stability of 4.0- and 6.5-mm cannulated partially threaded screws with round washers. Despite the absence of a washer, the 5.0 headless cannulated compression screws also demonstrated a surprisingly high stiffness and low displacement as compared to the other screws. In contrast, the stability of plate fixation was disappointing. None of the plates was able to tolerate loads above 100 N. In terms of overall stability, the best results were observed with 6.5-mm cannulated partially threaded screws, followed by 5.0 headless cannulated compression screws and 4.0-mm cannulated partially threaded screws, respectively. Plate fixation was found to be significantly weaker. In calcaneal beak fractures, the skin and soft tissue cover over the heel is at risk and should be handled with great caution (Banerjee et al., 2012; Gitajn et al., 2015; Mitchell et al., 2019). The advantage of the percutaneous operative technique is the very low risk of soft tissue trauma compared to open techniques. The screw fixation methods presented here allow percutaneous fixation with minimal soft tissue irritation. When using conventional screws, the screw head and washer can be placed at the level of the Achilles tendon insertion, usually without interfering with the surrounding tissue. From a clinical point of view, the risk of soft tissue irritation can be further reduced through the use of the headless cannulated compression screws, which enables the burial of the screw head in the bone. This method may also reduce the necessity of implant removal. However, screw revision may be more difficult due to the challenge of finding the screw head under the bone level. But the clinical advantage of these headless cannulated compression screws requires evaluation through clinical studies and cannot be confirmed by our biomechanical study. In addition, in cases where the fragment does not adapt well, conversion to open exposure and fixation may become necessary. In these and in revision cases, plate fixation may be an option. To our knowledge, no biomechanical study has been conducted regarding the use of 2.3- or. 2.8-mm plates. As mentioned previously, our results raise concerns about the fixation of beak-type fractures using plate osteosynthesis. The stability is inferior compared to screws. In cases where plates are used for surgical revision, limited postoperative mobilization is vital. Despite our poor biomechanical results, successful reports of plate fixation for beak fractures exist (Agni and Fearon, 2016). A combination of plate and screw fixation is also possible (Yu et al., 2013).

**FIGURE 7:**
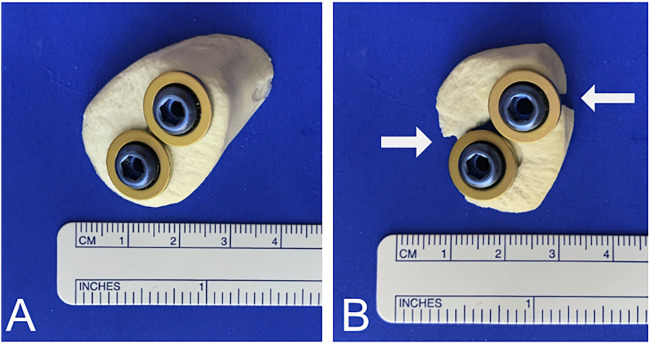
Screw-to-bone ratio. **(A)** To avoid burst of the fracture caused by oversized screws, a 1:2 screw-to-bone ratio is recommended. **(B)** Cracks and bursts of the fracture can occur when the screw size is not appropriate. White arrows indicate fracture.

Careful selection of screw sizes is vital and screw size is limited by the dimensions of the fragment. To avoid an iatrogenic burst of the bony fragment through the use of inadequate screws, we recommend a screw-bone ratio of roughly 1:2 (Figure 7). Although suture anchors were not included in the test protocol, they may also be used to augment screw fixation for bony fragments (Khazen et al., 2007; Yoshida et al., 2016).

**FIGURE 8:**
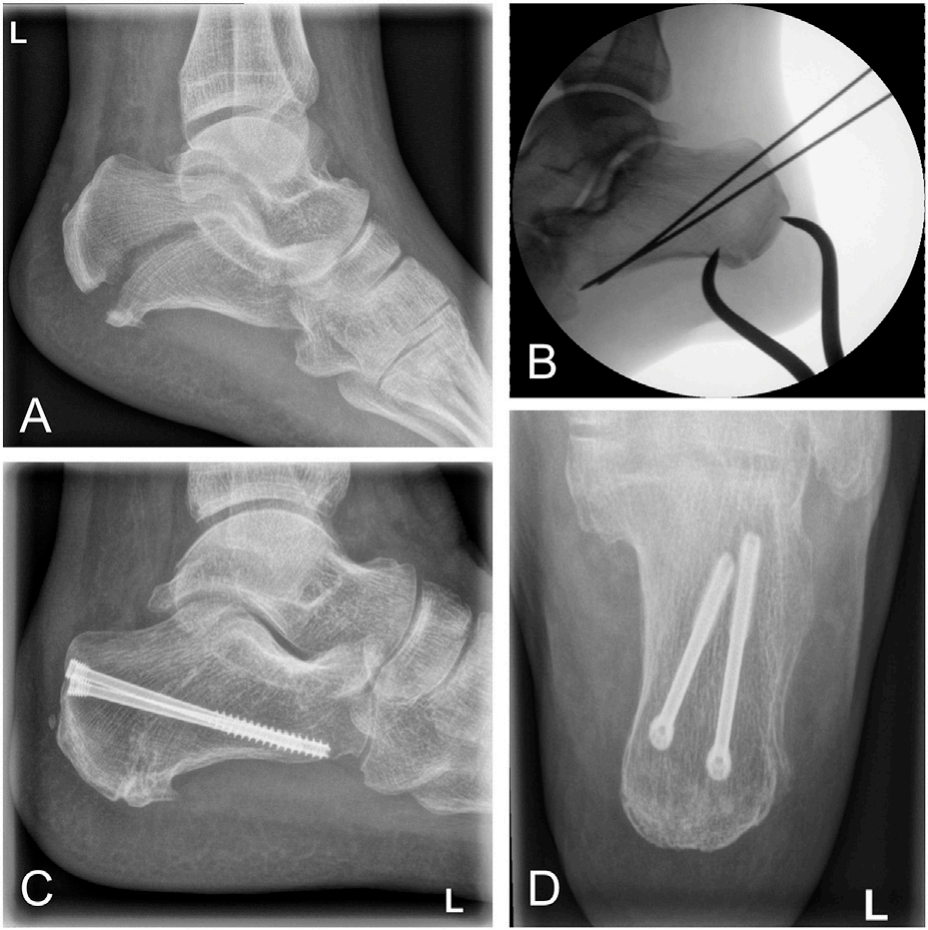
Clinical case of a calcaneal avulsion fracture successfully treated using 5.0-mm headless cannulated compression screws. **(A)** Lateral x-ray showing a fracture gap caused by tension of the Achilles tendon. **(B)** Intraoperative fluoroscopy demonstrating percutaneous reduction by a pointed reduction clamp. **(C** and **D)** Postoperative x-ray after 12 months demonstrating osseous healing in lateral and ap views.

**FIGURE 9:**
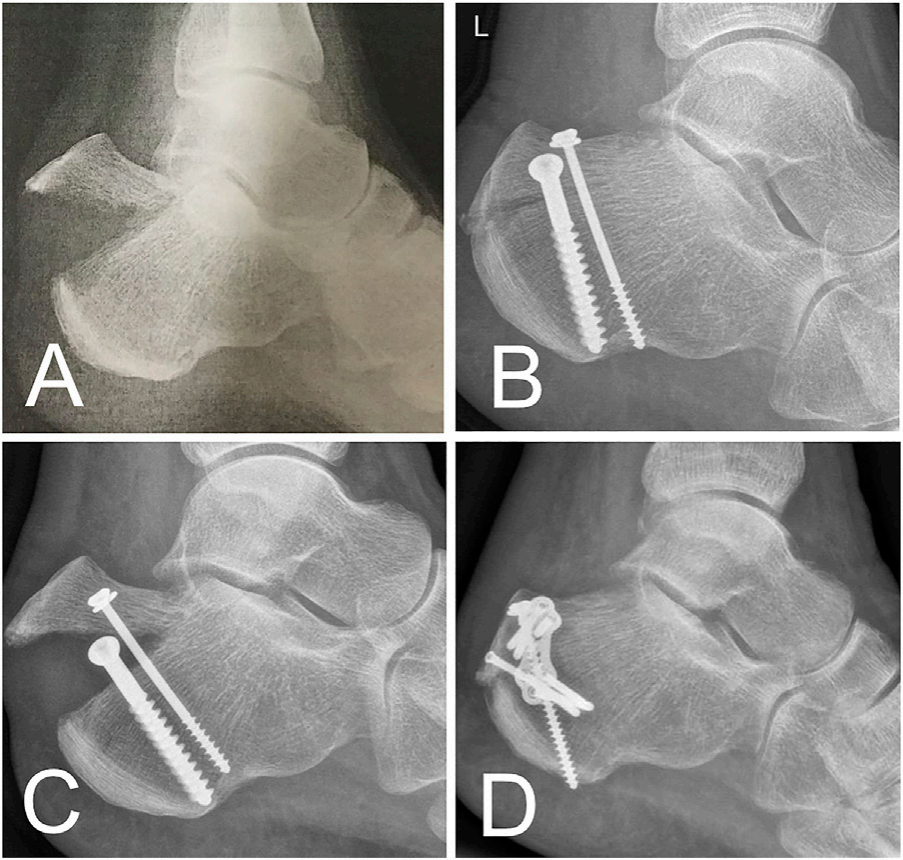
Clinical case. **(A)** Beak fracture characterized by a solid bone part in a 61-year-old female patient. **(B)** X-ray after urgent reduction and screw fixation. **(C)** Early fracture dislocation after mobilization of the patient. **(D)** Revision performed using a combination of screw and plate osteosynthesis.

Clinical cases further underline our findings. Based on the results of this biomechanical study, we implemented headless cannulated compression screws in a clinical case of a calcaneal avulsion fracture with critical soft tissue findings. The fracture healed completely, and the clinical function was excellent after 6 months (Figure 8). Despite this success, an open exposure and visually controlled reduction to ascertain anatomic fixation of the fragment may be necessary for more complex fractures and may be superior to percutaneous fixation (Banerjee et al., 2012; Blum et al., 2019). In another clinical case, a screw cut-out caused a re-dislocation of the fragment. The use of an additional plate enabled the fixation of a fragment too small for 6.5-mm or 4.0-mm diameter screws. The fracture healed well following the first revision using a restrictive postoperative motion protocol (Figure 9). Despite this, we do not recommend plating of beak fractures as a first-line treatment.

Despite this, results for the most promising fixation techniques require follow-up confirmation in a biomechanical setting with cadaver specimens. Furthermore, while fixation of the tension band on the tuber calcanei was challenging, the simple test set-up does not mimic the properties of *in vivo* Achilles tendons. This became most obvious under high loads. Load vectors may also be different under real-life conditions. In addition, the small proportions of the plates used may be responsible for their poor biomechanical performance compared to screws. Despite these limitations, this study represents the largest biomechanical study to date regarding this uncommon injury.

## Conclusion

1) Generally, 6.5-mm partially threaded screws and 5.0-mm headless cannulated compression screws have the best overall stability for beak fracture fixation.

2) Whenever the fragment size does not allow one of the screws mentioned earlier, two 4.0-mm partially threaded screws are a good alternative.

3) In general, 2.3-mm or 2.8-mm bend plates cannot be recommended. If used, a combination of screws, suture anchors, or other fixation techniques and a very restrictive postoperative rehabilitation protocol is recommended.

4) Screw cut-out is mode-of-failure in partially threaded screws. Fragment pull-out occurred in 5.0-mm headless cannulated compression screws. Breakout of the fragment happens when plates are used.

## Data Availability

The original contributions presented in the study are included in the article/Supplementary Material; further inquiries can be directed to the corresponding author.
